# Supplementary material for: A sampling survey of enterococci within pasteurized, fermented dairy products and their virulence and antibiotic resistance properties
Source: PLoS One. 2021 Jul 15;16(7):e0254390. doi: 10.1371/journal.pone.0254390 (PMC8282027; doi:10.1371/journal.pone.0254390)
Supplement: S1 Fig — PCR results for Enterococcus identification (A) and VF detection (B, efaA; C, cpd, ccf; D and E, cob). PCR for species identification of Enterococcus. Upper panel: Lane 1: Molecular weight marker (MWM) (1 Kb Plus), 2: Isolate 1, 3: Isolate 21, 4: Isolate 22, 5: Isolate 2, 6: Isolate 62, 7: Isolate 63, 8: Isolate 51, 9: Isolate 52, 10: Isolate 3, 11: Isolate 4, 12: Isolate 5. Lower panel: Lane 1: MWM (1 Kb Plus), 2: Isolate 61, 3: Isolate 45, 4: Isolate 6, 5: Isolate 25, 6: Isolate 30, 7: E. faecalis (ATCC), 8: Isolate 32 (a cheese isolate), 9: Negative control. Isolates 45, 25, and 30 were run for the species PCR again with increased primer/template combinations and found to be E. faecium (data not shown). (B) PCR for VF detection (efaA). Upper panel: Lane 1: MWM (1 Kb Plus), 2: E. faecalis (ATCC), 3: Isolate 1, 4: Isolate 2. 5: Isolate 3, 6: Isolate 4, 7: Isolate 5, 8: Isolate 6, 9: Isolate 21, 10: Isolate 22: 11: Isolate 25, 12: Isolate 30. Lower panel: Lane 1: MWM (1 Kb Plus), 2: E. faecalis (ATCC), 3: Blank lane, 4: Isolate 45, 5: Isolate 51, 6: Isolate 52, 7: Isolate 61, 8: Isolate 62, 9: Isolate 63, 10: Negative control. Isolates 51, 62 and 63 were found to contain efaA in a different optimized PCR run. (C) PCR for VF detection (ccf and cpd). Upper panel: Lane 1: MWM (1 Kb Plus), 2: E. faecalis (ATCC) (with both cpd and ccf). 3: Isolate 1, 4: Isolate 2, 5: Isolate 3, 6: Isolate 4, 7: Isolate 5, 8: Isolate 6, 9: Isolate 21, 10: Isolate 22: 11: Isolate 25, 12: Isolate 30. Lower panel: Lane 1: MWM (1 Kb Plus), 2: E. faecalis (ATCC), 3: Blank lane, 4: Isolate 45, 5: Isolate 51, 6: Isolate 52, 7: Isolate 61, 8: Isolate 62, 9: Isolate 63, 10: Negative control. Isolates 61 and 63 were found to contain both ccf and cpd in a different PCR. (D) PCR for VF detection (cob). Upper panel: Lane 1: MWM (1 Kb Plus), 2: E. faecalis (ATCC), 5: Isolate 4, 6: Isolate 5. Lower panel: Lane 1: MWM (1 Kb Plus), Lane 6: Isolate 62. (E) PCR for VF detection (cob) for additional isolates [file pone.0254390.s001.pdf]

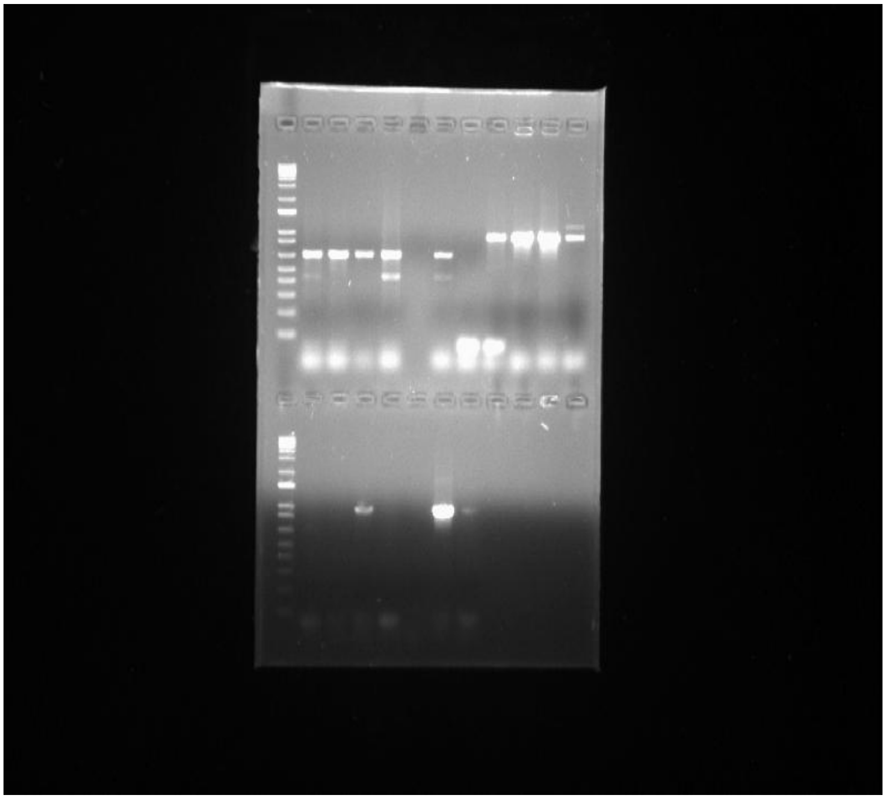

A

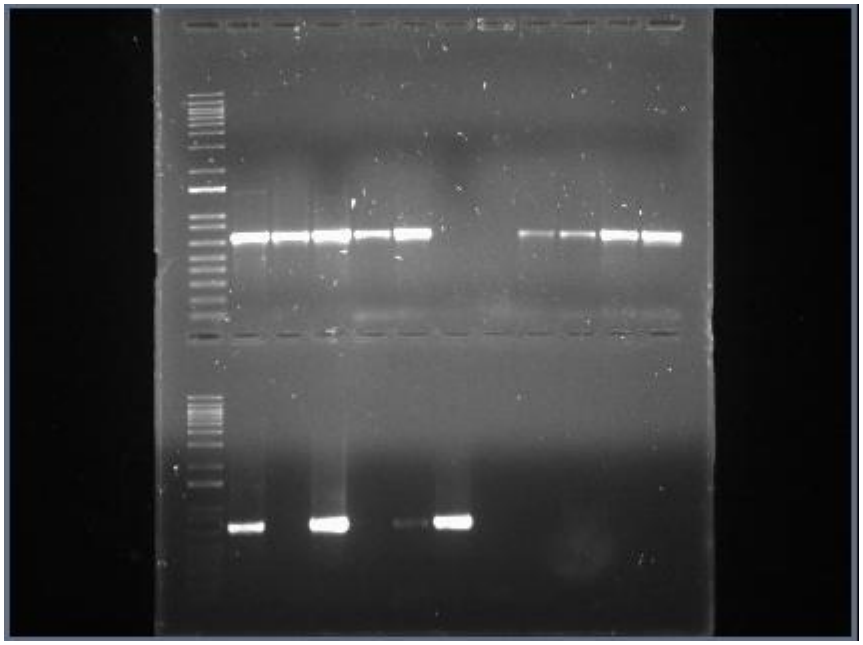

B

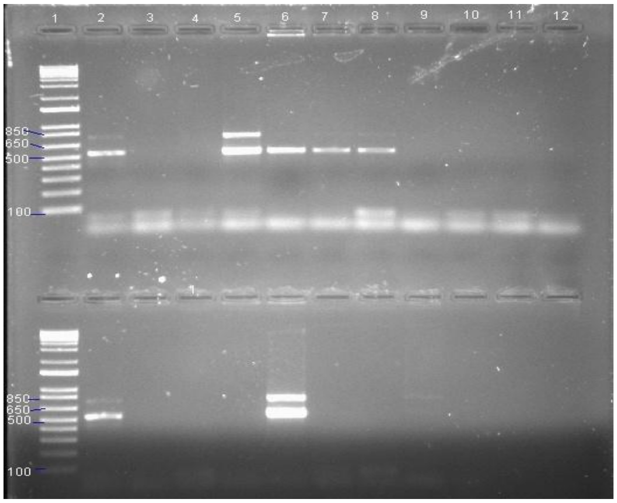

C

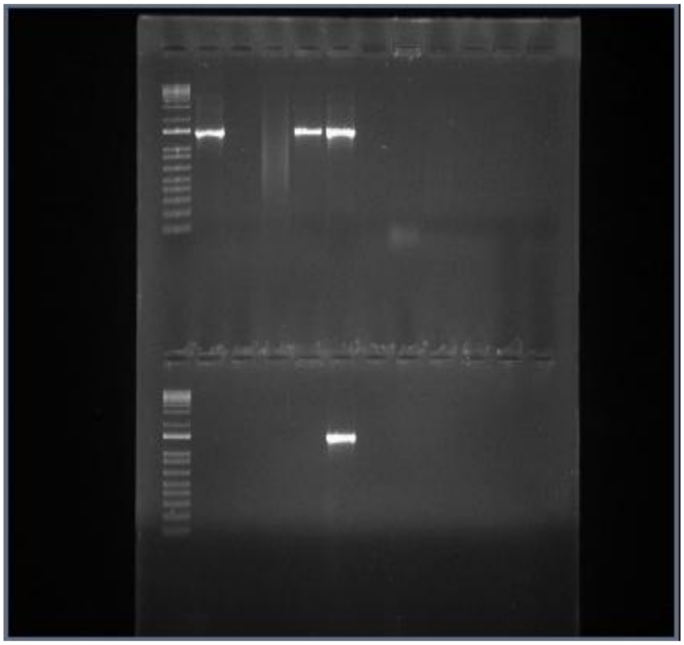
D

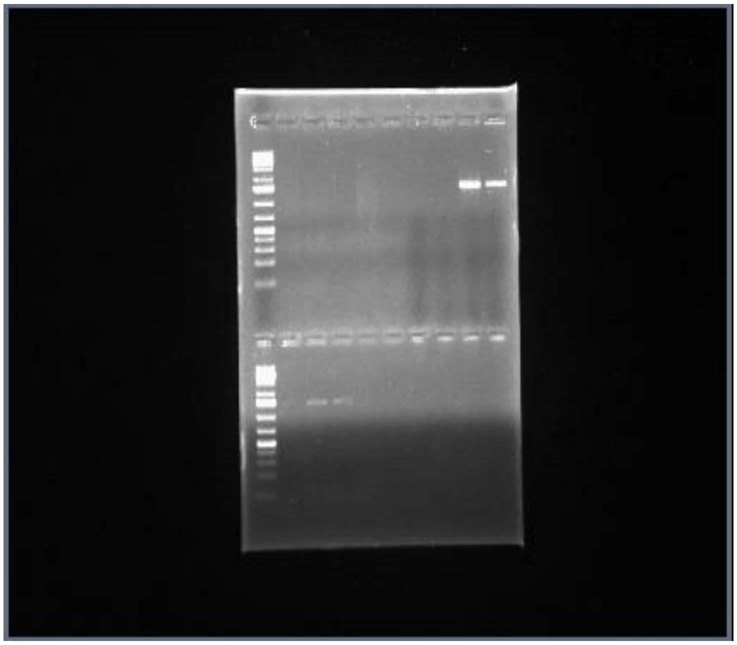

E

S1 Fig. (A-E) **PCR results for *Enterococcus* identification (A) and VF detection (B, *efaA*; C, *cpd*, *ccf*; D and E, *cob*).**
