# Supplementary material for: A sampling survey of enterococci within pasteurized, fermented dairy products and their virulence and antibiotic resistance properties
Source: PLoS One. 2021 Jul 15;16(7):e0254390. doi: 10.1371/journal.pone.0254390 (PMC8282027; doi:10.1371/journal.pone.0254390)
Supplement: S1 Table — (PDF) [file pone.0254390.s002.pdf]

Product	Status, size	Date purchased(D/M/Y)	Expiry date	Enterococcus found?	Product code**
Pasteirizes cream	refrigerated, 500 mL	02/03/17	04/03/17	No*	
Yogurt	refrigerated, 500 mL	02/03/17	11/03/17	Yes	
Cheese	frozen, 500g	02/03/17	not indicated	Yes	
Yogurt	refrigerated, 200 mL	02/03/17	06/03/17	Yes	
Milk	refrigerated, 5-- mL	02/03/17	11/03/17	Yes	
Cheese	refrigerated, 500g	10/02/20	not indicated	Yes	2
Cheese	refrigerated, 500g	10/02/20	not indicated	No*	3
Yogurt	refrigerated, 250 mL	10/02/20	18/02/20	Yes	7
Cheese	refrigerated, 500g	16/12/20	not indicated	No*	4
Cheese	refrigerated, 500g	16/12/20	not indicated	No*	5
Yogurt	refrigerated, 250g	16/12/20	26/12/20	No*	8
Milk	refrigerated, 500 mL		not indicated	Yes	1
Yogurt	refrigerated, 500 mL		not indicated	NT	6
Yogurt	refrigerated, 150 mL	16/12/20	24/12/20	NT	
National collection	Frozen stocks of already isolated bacteria (8) were donated for this study				

* - It is possible enterococci would still be found; it is only among the few randomly-selected colonies that enterococci were not found.

** - Product code is for those for which viable counts (CFU) were performed. No batch/lot numbers were available on product package..

NT – Not tested.
