# Supplementary material for: A sampling survey of enterococci within pasteurized, fermented dairy products and their virulence and antibiotic resistance properties
Source: PLoS One. 2021 Jul 15;16(7):e0254390. doi: 10.1371/journal.pone.0254390 (PMC8282027; doi:10.1371/journal.pone.0254390)
Supplement: S2 Table — (PDF) [file pone.0254390.s003.pdf]

S2 Table. Phenotypic-genotypic tests of bacterial isolates.

Source/Isolate*	GS	Ca	O	BE	Na (6.5%)	45°C	10°C	60°C	BA	Gelatinase	PYR	Identity (presumed or PCR)
				Growth in/at								
**Yogurt**												
1	Co	–	–	+	G	G	G	NG	β	–	+	*E. fm*
2	Co	–	–	+	G	G	G	NG	β	–		*E. fm*
3[϶]	Co	–	–	+	G	NG	G	NG	α	–	–	*E. fs*
4[϶]	Co	–	–	–	G	G	G	NG	β	–	+	*E. fs*
5[϶]	Co	–	–	–	G	NG	G	G	β	–	–	*E. fs*
6[϶]	Co	–	–	–	G	NG	G	NG	α	–	–	*E. fs*
7-14[Ω]	GN	+	+									GN rods
15-19[Ψ]	Co	–		+	G							Non-E LAB
**Cheese**												
21[δ]	Co	–	–	+	G	G	NG	NG	β	–	+	*E. fm*
22[δ]	Co	–	–	+	G	G	G		α	–	+	*E. fm*
23[µ]			–	G						–		Unidentified
24[µ]				G						–		Unidentified
25[π]	Co	–		–	G	NG	NG	NG	α	–		*E. fm*
26[π]	Co	–			NG	G	G	NG	α	–	–	NE-LAB
27[π]	Co	–	+	+	G	NG	NG	NG	α	–		GN
28[π]	Co	–	+	+	G	G	G	NG		–	–	GN
29[π]	Co	–			G	G	G	NG		–		NE-LAB
30[π]	Co	–	–	+	G	G	G	NG	*β*	–	–	*E. fm*
31[π]	Co	–					G					NE-LAB
32[π]	Co	–		–	G	G	G	NG	*β*	–		NE-LAB
33[θ]	GN	+			G							G- rods
34-37[θ]	GN	+			G							Yeasts
38[ϕ]	Co	+			NG							GN
39-43[ϕ]		+			G							Yeasts[@]
44[ϕ]	Co	+			G							Probable *S.aureus*(GP)

Supplementary Table 1 cont'd

Source/Isolate[*]	GS	Ca	O	BE	Na (6.5%)	45°C	10°C	60°C	BA	Gelatinase	PYR	Identity (presumed or PCR)
				Growth in/at								
**Milk**												
45[κ]	Co	–	–	NG[##]	G	NG			γ	–	–	*E. fm*
46[κ]	Co	–	–	NG[##]	G	NG				–	–	NE-LAB
47-49[η]	GN											GN rods
50[⊥]	Co	–	–	–	+	–			γ			NE-LAB
51[⊥]	Co	–	–									*E. fm*
52[⊥]	Co	–	–									*E. fs*
53	Co	+	+	NG	NG	NG	G	G	NG	–	–	GN
54-55[χ]	GN–	–	–	NG	–	G		G	γ	–		GN
56[χ]	GN	–	–	+	–				α			GN
57[χ]	GN	+	+									GN
**58	GN	+	+		G	G		G		–		GN
59-60[ν]	Co	–	–									NE-LAB
**National Collection**												
61	Co	–	–	+	G	G			γ	–	+	*E. fm*
62	Co	–	–	+	G	G			α	–	+	*E. fm*
63	Co	–	–	+	G	G			α	–	–	*E. fm*
64	Co	–	–	–	G	G			α	–	–	NE-LAB
65-66	GN	+										GN
67	Co	+	–	–	G	NG			β	–	–	GN
68	Co	+		NG[##]	G	G			β	–	–	GN
**Cream**												
69	Co	–	–	+	G	G	NG		α	–	+	Non-E LAB

Supplementary Table 1 continued…

Source/Isolate[*]	GS	Ca	O	BE	Na (6.5%)	45°C	10°C	60°C	BA	Gelatinase	PYR	Identity
					Growth in/at							
E. faecalis		–	–	+	G	G	G		β	–	+	Type strain
S. aureus		+							β	–		Type strain
E. coli			–	–/NG	NG			–				Type strain
S. pyogenes									β			Type strain
P. aeruginosa			+									Type strain

Abbreviations and symbols used: GS: Gram stain; Ca: catalase; O: oxidase; Co: cocci; G: growth; NG: nogrowth; GN: gram-negative; GP: gram-positive; +: positive; –[##] - this isolate was inhibited on BE (bile-esculin)-containing medium (this is indicated as NG); @ - No rods, no cocci - seemed yeast presence was detrimental to bacteria; Non-E LAB: non-*E. faecalis*, non-*E. faecium* LAB.

Isolates with the same superscript symbol are multiple independent isolates from the same product. Others are independent isolates from different products, except the National collection, the source of which are various foods (but they are not clinical or animal isolates).
