# Supplementary material for: A sampling survey of enterococci within pasteurized, fermented dairy products and their virulence and antibiotic resistance properties
Source: PLoS One. 2021 Jul 15;16(7):e0254390. doi: 10.1371/journal.pone.0254390 (PMC8282027; doi:10.1371/journal.pone.0254390)
Supplement: S3 Table — (PDF) [file pone.0254390.s004.pdf]

S3 Table. Disk Diffusion Test Results for the study isolates.

Isolate	Antibiotic						
	ERY	TET	AMP	OXC	CPR	AZM	VAN
2	R	R	R	R	I	R	R
3	R	R	R	R	I	R	R
21	R	R	R	R	I	R	R
30	R	R	R	R	I	R	S
31	R	S	R	11	I	R	S
45	R	I	R	R	S	R	R
S. au	I	I	S	S	I	I	R
E. coli	I	S	S	S	I	I	
E. fec	R	S	S	R	I	R	R

Abbreviations used: ERY - erythromycin, TET – tetracycline, AMP - ampicillin, OXC – oxacillin, CTR – ceftriaxone, CRX – cefuroxime, CPR - ciprofloxacin, NAL – nalidixic acid, AZM – azithromycin, VAN – vancomycin, R – resistant, I – intermediate, S – susceptible. Interpretive criteria for ceftriaxone, cefuroxime and nalidixic acid that were included in this test were not found in the CLSI guideline.
