# Supplementary material for: A sampling survey of enterococci within pasteurized, fermented dairy products and their virulence and antibiotic resistance properties
Source: PLoS One. 2021 Jul 15;16(7):e0254390. doi: 10.1371/journal.pone.0254390 (PMC8282027; doi:10.1371/journal.pone.0254390)
Supplement: S4 Table — (PDF) [file pone.0254390.s005.pdf]

S4 Table. **Susceptibility test results for selected isolates using VetMIC Plate (VetMIC Lact2) assay Plate format is shown below.**

<u>**Isolate**</u>	<u>**Susceptibility test results**</u>
2	Growth in all 96 wells, but some growth reduction in VAN wells C8-C11, VAN MIC ≥ 16. µg/mL
3	Growth in all 96 wells
4	Growth in all wells except Van wells 7-11 where there was much reduced growth, VAN MIC ≥ 8 µg/mL.
5	Growth in all 96 wells.
32	Growth in all 96 wells, except some growth reduction in VAN wells C9-C11 compared to the lower concentration wells, VAN MIC ≥ 32 µg/mL.
E. faecalis	Growth in all wells, except VAN C8-C11, VAN MIC ≥ 16 µg/mL.

<u>**VetMIC Lact-2** (version 2014-06) Art. E395115</u>

Panel for susceptibility testing of bacteria. 100µL/well gives concentrations (µg/mL) as below

**Lact-2**	**1**	**2**	**3**	**4**	**5**	**6**	**7**	**8**	**9**	**10**	**11**	**12**
**A**	P	**Am** 0.03	0.06	0.12	0.25	0.5	1	2	4	8	16	N
**B**	P	**Pc** 0.03	0.06	0.12	0.25	0.5	1	2	4	8	16	N
**C**	P	**Va** 0.25	0.5	1	2	4	8	16	32	64	128	N
**D**	P	**Qda** 0.016	0.03	0.06	0.12	0.25	0.5	1	2	4	8	N
**E**	P	**Lz** 0.03	0.06	0.12	0.25	0.5	1	2	4	8	16	N
**F**	P	**Tm** 0.12	0.25	0.5	1	2	4	8	16	32	64	N
**G**	P	**Ci** 0.25	0.5	1	2	4	8	16	32	64	128	N
**H**	P	**Ri** 0.12	0.25	0.5	1	2	4	8	16	32	64	N

P: The positive control wells contain the diluent used with the corresponding antimicrobial agent. N: The negative control wells are empty.

Am	Ampicillin	Lz	Linezolid
Pc	Penicillin	Tm	Trimethoprim
Va	Vancomycin	Ci	Ciprofloxacin Ri Rifampin
Qda	Quinupristin-dalfopristin	Ri	Rifampin
